# Prospective Study About the Relationship Between CEUS of Carotid Intraplaque Neovascularization and Ischemic Stroke in TIA Patients

**DOI:** 10.3389/fphar.2019.00672

**Published:** 2019-06-21

**Authors:** Zhenzhou Li, Xianfeng Xu, Lijie Ren, Yufeng Shao, Shuyu Luo, Shenghua Chen, Xiaoyun Guan

**Affiliations:** ^1^Department of Ultrasound, The Second People’s Hospital of Shenzhen, The First Affiliated Hospital of Shenzhen University, Shenzhen, China; ^2^Department of Obstetrics and Gynecology, The Second People’s Hospital of Shenzhen, The First Affiliated Hospital of Shenzhen University, Shenzhen, China; ^3^Department of Internal Neurology, The Second People’s Hospital of Shenzhen, The First Affiliated Hospital of Shenzhen University, Shenzhen, China

**Keywords:** contrast-enhanced ultrasonography, carotid plaque, neovascularization, transient ischemic attack, ischemic stroke

## Abstract

**Objective:** To evaluate the relationship between contrast-enhanced ultrasonography (CEUS) of carotid intraplaque neovascularization and ischemic stroke in transient ischemic attack (TIA) patients.

**Methods:** A total of 112 TIA patients were selected for the study. Routine carotid ultrasonic examination was performed for all the patients. CEUS was carried out for consecutive patients with plaque thicker than 2.5 mm in carotid bifurcation and follow-up for at least 24 months. The number of patients with incurrence of ischemic stroke or recurrence of TIA was obtained during the follow-up period. To detect the risk factors for incurrence of ischemic stroke or recurrence of TIA in 24 months, multivariate logistic regression analyses were performed for all the risk factors in all the selected patients.

**Results:** Ninety-one patients underwent CEUS and were followed up at least 24 months. There were statistical differences between recurrent and non-recurrent groups about hypertension, diabetes, hyperlipemia, smoking history, family history of stroke, medication compliance, two-dimensional ultrasound, and CEUS (*P* < 0.05). The higher CEUS intensity in the carotid plaque was, the higher was the possibility of ischemic stroke or recurrent TIA. Multivariate logistic regression analysis showed that the CEUS characteristics of carotid plaque such as linear enhancement or diffuse enhancement were independent risk factors for ischemic stroke or recurrent TIA in TIA patients (*P* < 0.05).

**Conclusion:** For carotid plaques, CEUS could evaluate the infusion mode, which could reflect the neovascularization in plaques. CEUS could predict the incurrence of ischemic stroke or recurrence of TIA in TIA patients, which is useful information when making a clinical decision.

## Background

Transient ischemic attack (TIA) is a brief and reversible episode of neurological dysfunction disorders. It is an important risk factor for cerebral infarction. Studies (Wu et al., 2007; Uehara et al., 2017) showed that the incidence of cerebral infarction after TIA ranged from 8.0% to 10.5%. Carotid arteries are essential arteries that supply the brain. Plaques building up inside the arteries are one of the leading causes of TIA and cerebral infarctions (Parmar et al., 2010; Sun et al., 2018). Some features of carotid plaque were mostly associated with the instability of plaque, including intraplaque hemorrhage, lipid-rich necrotic core, and surface disruption, and the instability of plaque is believed to be the main reason for the occurrence of ischemic strokes. Assessing plaque stability is one of the major objectives in the etiological study of ischemic stroke (Ott et al., 2008). With conventional ultrasound, stability can be estimated from the size, morphology, and echogenicity of the plaques, and it has a relatively high specificity in predicting the risk of ischemic stroke (Reiter et al., 2008). It is found that intraplaque neovascularization contributes to the instability of plaques (van Hoof et al., 2017).

A high signal intensity on T1-weighted magnetic resonance imaging (MRI) is a definitive finding indicating the vulnerability of carotid plaques (Hosseini et al., 2013). The addition of MRI to the pathological findings can confirm the vulnerability of carotid-artery plaques (Yuan et al., 2001). Contrast-enhanced carotid ultrasound (CEUS) has been shown to correlate with the MRI and pathological findings of carotid plaque, which can be used to evaluate intraplaque vessels and the vasa vasorum (Shimada et al., 2018) and which correlates significantly with the frequency of cardiovascular risk factors and the development of cardiovascular disease (Staub et al., 2010).

Thus, there is a need to further understand whether perfusion of intraplaque neovascularization is an independent risk factor for ischemic stroke or recurrent TIA. The aim of this study was to evaluate the perfusion mode of the intraplaque neovascularization by CEUS for TIA patients and investigate the correlation between the perfusion mode of carotid plaques and the risk of ischemic stroke or recurrent TIA.

## Methods

### Study Population

A total of 112 TIA patients with carotid plaques were prospectively recruited at the Stroke Unit, Shenzhen No. 2 People Hospital between January 2014 and February 2016. The inclusion criteria were as follows: 1) Patients had a past history of TIA. According to the guidelines of the American Stroke Association (ASA) in 2009, TIA was defined as a brief episode of neurological dysfunction resulting from focal cerebral or retinal ischemia, with clinical symptoms typically lasting less than 1 h, and without evidence of acute infarction on imaging. 2) Carotid plaques were determined by conventional ultrasound with plaque thickness ≥2.5 mm. 3) Age >45 years. The exclusion criteria were as follows: 1) Patients had a history of cerebral vascular diseases such as ischemic and hemorrhagic infarctions. 2) Patients were disoriented and confused, or they could not follow the doctor’s commands. 3) Patients had severe infections, malignant tumors, cardiopulmonary dysfunction, hepatorenal failure, or respiratory failure. 4) Plaques were mainly hyperechoic or uniformly hyperechoic. 5) Patients refused to undergo CEUS examination. 6) Patients were lost to follow-up.

One hundred and twelve patients were enrolled in this study; 17 patients did not undergo CEUS and follow-up return visits, owing to their plaques being mainly hyperechoic or uniformly hyperechoic, which means that the majority of or the whole plaque is calcification, which is difficult for CEUS. Four patients were lost follow-up. Ninety-one patients underwent CEUS and were followed up at least 24 months, of whom 41 were men and 50 were women. Age ranged from 48 to 88 years (median age, 67.8 ± 10.3 years). Subjects included 49 patients with hypertension, 59 patients with diabetes mellitus, 37 patients with hyperlipidemia, 39 patients with a history of smoking, 30 patients with a family history of stroke, and 33 patients with poor medication adherence. During at least 24 months of follow-up, 13 patients suffered an ischemic stroke and 24 patients had a recurrent TIA. All of the patients gave their written informed consent. Approval for this study was obtained from the institutional ethics committee of Shenzhen No. 2 People’s Hospital.

### Imaging Protocol

Subjects were imaged using a Siemens Acuson S2000 ultrasound platform equipped with contrast pulse sequence (CPS) software. A 9L4 linear probe (7–14 MHz) was used for ultrasound examinations. Subjects were asked to lie supine with head rotated away from the side being examined. After the longitudinal and transverse scanning of the common carotid arteries was done, the size, location, and intraplaque echogenicity of the plaques were intensively recorded. Common carotid or carotid bifurcation plaques thicker than 2.5 mm were selected for CEUS examination. For subjects with multiple plaques, only the largest plaque was selected. With reference to the echogenicity of adjacent sternocleidomastoid muscles, carotid plaques were defined as relatively hyperechoic, isoechoic, or hypoechoic. Features of instability including an ulcerated or irregular surface contour, discontinuous intima echo, eccentric index >2, and intraplaque liquefaction were marked.

**Figure 1:**
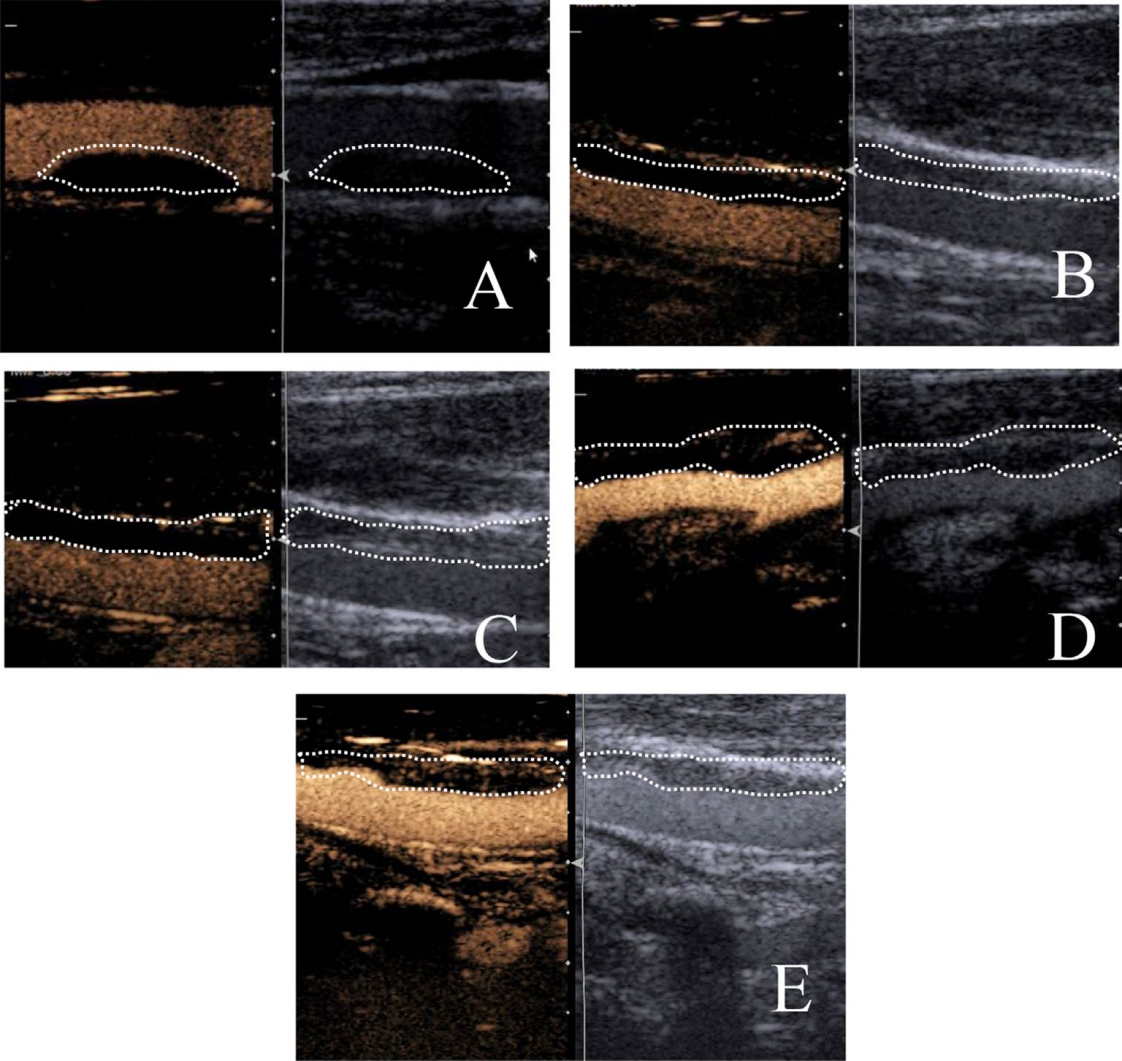
Grading of the intraplaque contrast enhancement. **(A)** Grade 0: no enhancement; **(B)** Grade 1: adventitia of arterial wall enhancement, no intraplaque enhancement; **(C)** Grade 2: intraplaque spot enhancement; **(D)** Grade 3: linear enhancement that extends into the plaque; **(E)** Grade 4: intraplaque diffuse enhancement (left: plaques before contrast enhancement; right: plaques after contrast enhancement).

After obtaining a satisfying image of the selected plaques, CEUS was turned on. The examination mode was then switched to cadence-CPS and the imaging was performed with a mechanical index of 0.2. A bolus of 2.5 ml of Sonovue suspension was injected into the median cubital vein of the left arm. Once the contrast agent had been injected, and the Intravenous access was flushed with 5 ml of 0.9% sodium chloride. The recording of the image was started as the contrast was injected. The filling process of contrast agents within the vessel lumen and the plaques were illustrated. In order to evaluate the intraplaque contrast enhancement, the ultrasonologists only took into account the dynamic spot hyperechoic lesions within the plaques or the border of the plaques (Coli et al., 2008). Grading of the intraplaque contrast agent enhancement followed the classification of Lee (Li et al., 2013): Grade 0: no enhancement; Grade 1: adventitia of arterial wall enhancement, no intraplaque enhancement; Grade 2: intraplaque spot enhancement; Grade 3: linear enhancement that extends into the plaque; Grade 4: intraplaque diffuse enhancement (**Figure 1**). Grading of the enhancement was completed by two trained and experienced doctors. When there was difference in grading between the two doctors, they discussed and determine the final result together.

### Follow-Up

After ultrasound examination, subjects obtained routine treatments to reduce risk factors for cardiovascular diseases such as hypertension, diabetes mellitus, and hyperlipidemia. A follow-up evaluation should be conducted every 3 months for at least 24 months. During the follow-up, patients were asked if they had an ischemic stroke or recurrent TIA, and if they took medication as prescribed. Medication adherence was measured by the proportion of days when medications were taken as prescribed over the follow-up. A threshold of 80% was used to classify patients as adherent or nonadherent (Cramer et al., 2008). If a patient in the study could follow the doctor’s orders to take medication greater than or equal to 80% days in the study period (24 months), we think that the patient is adherent; otherwise, the patient will be classified as nonadherent. Ischemic stroke was defined as a persistent neurological deterioration that occurred after TIA was resolved, accompanied by neuroimaging evidence. TIA was defined as a new onset neurological functional deficit with symptoms resolved within 1 h (Fang et al., 2013). After follow-up, subjects who had ischemic stroke or recurrent TIA were regarded as the “recurrent” group and those who did not have ischemic stroke or recurrent TIA were regarded as the “non-recurrent” group.

### Statistical Analysis

Statistical analysis was performed using SPSS Statistics 17.0 software. Frequency and percentages (%) were used for categorical variables, whereas mean ± SD were employed for continuous variables. Continuous variables were compared between two groups using the independent *t* test. Proportions were compared using the chi-square test. To determine the association between variables and ischemic stroke or recurrent TIA after TIA, a multivariate logistic regression analysis was performed. Receiver operation curve (ROC) was generated to examine the efficacy of the resulting model. The level of significance was set at *P* < 0.05.

### Inter-Observer Variability

A total of 20 plaques with CEUS were selected, and the CEUS grade of the intraplaque contrast agent enhancement was judged by two blinded, experienced doctors. If the CEUS grade judgments of the two doctors were the same, we recorded it as uniformity; otherwise, we recorded it as nonuniformity. The inter-observer variability was expressed as percentile differences: the number of plaques recorded as nonuniformity/20 × 100%.

## Results

Of 91 patients who were included in this study, 37 were in the “recurrent” group, while 54 were in the “non-recurrent” group. There was no statistical difference in terms of age and sex (*P* > 0.05). **Table 1** shows the intergroup difference in history of hypertension (*P* = 0.001), diabetes (*P* = 0.025) or hyperlipidemia (*P* = 0.001), history of smoking (*P* = 0.027), the family history of cerebral stroke (*P* = 0.029), and medication adherence (*P* = 0.001).

**Table 1:** Intergroup comparisons of patient characteristics.

Group	Age (years)	Sex (M/F, *n*)	Hypertension (Y/N, *n*)	Diabetes mellitus (Y/N, *n*)
Recurrent (*n* = 37)	70.1 ± 10.9	16/21	28/9	29/8
Non-recurrent (*n* = 54)	66.3 ± 9.6	25/29	21/33	30/24
*t* or χ^2^	1.735	0.083	11.956	5.016
*P*	0.086	0.774	0.001	0.025
Group	Hyperlipidemia (Y/N, *n*)	Smoking (Y/N, *n*)	FHx of cerebral stroke (Y/N, *n*)	Medication adherence (Y/N, *n*)
Recurrent (*n* = 37)	23/14	21/16	17/20	16/21
Non-recurrent (*n* = 54)	14/40	18/36	13/41	42/12
*t* or χ^2^	11.949	4.919	4.753	11.329
*P*	0.001	0.027	0.029	0.001

Of 91 carotid plaques selected, 33 were isoechoic, 27 were hypoechoic, and 31 were mix-echoic. The maximal thickness of plaques was 3.2 ± 0.7 mm. Twenty-seven plaques were with signs of instability such as irregular or ulcerated surface contour, intraplaque liquefaction, and eccentric index >2. For contrast-enhanced plaques, 27 plaques were Grade 0, 10 were Grade 1, 23 were Grade 2, 20 were Grade 3, and 11 were Grade 4. **Table 2** illustrated the statistical difference between two groups in conventional ultrasonic features of instability and the grades of CEUS contrast enhancement (*P* < 0.05).

**Table 2:** Intergroup comparison of conventional ultrasonic features of instability and grade of CEUS (number of cases).

Group	Mode of ultrasound (stable/unstable, *n*)	CEUS (Grades 0–2/Grades 3–4, *n*)	Mode of ultrasound + CEUS (stable/unstable, *n*)
Recurrent (*n* = 37)	20/17	18/19	13/24
Non-recurrent (*n* = 54)	44/10	42/12	34/20
*t* or χ^2^	7.915	8.294	6.808
*P*	0.005	0.004	0.009

The data set consisted of one dichotomous dependent variable: prognosis coded 1 for the presence of ischemic stroke or the recurrence of TIA and 0 for no ischemic stroke or recurrent TIA. A multivariate logistic regression analysis was conducted. In the regression analysis, the presence of ischemic stroke and the recurrence of TIA were taken as dependent variables, and the eight independent variables whose intergroup differences were statistically significant (hypertension, diabetes mellitus, hyperlipidemia, smoking, family history of cerebral stroke, medication adherence, conventional ultrasonic features of instability, and grades of CEUS contrast enhancement) were taken as independent variables. **Table 3** shows the results from the multivariate regression analysis. Variables including family history of cerebral stroke, grade of CEUS in plaque, medication adherence, hypertension, conventional ultrasonic features of instability, and hyperlipidemia were independent risk factors of ischemic stroke and recurrent TIA. After adjusting for confounding effects, Grades 3–4 of CEUS contrast enhancement was found to be an independent risk factor to ischemic stroke or recurrent TIA (*P* < 0.05). These independent variables were further ranked according to the odds ratios, from highest to lowest: family history of cerebral stroke, grade of CEUS contrast enhancement, medication adherence, hypertension, conventional ultrasonic features of instability, and hyperlipidemia.

**Table 3:** Multivariate logistic regression analysis for ischemic stroke and recurrent TIA.

Variables	*R*	SE	Wald	*P*	OR	95% CI	
						Lower	Upper
Family history of cerebral stroke	2.592	0.801	10.483	0.001	13.354	2.781	64.127
Grade of CEUS contrast enhancement	1.919	0.747	6.600	0.010	6.817	1.576	29.484
Medication adherence	1.864	0.654	8.126	0.004	6.448	1.790	23.224
Hypertension	1.678	0.622	7.266	0.007	5.353	1.581	18.131
Conventional ultrasonic features of instability	1.557	0.696	5.010	0.025	4.747	1.214	18.565
Hyperlipidemia	1.234	0.604	4.176	0.041	3.434	1.052	11.208

OR, odds ratio.

**Figure 2:**
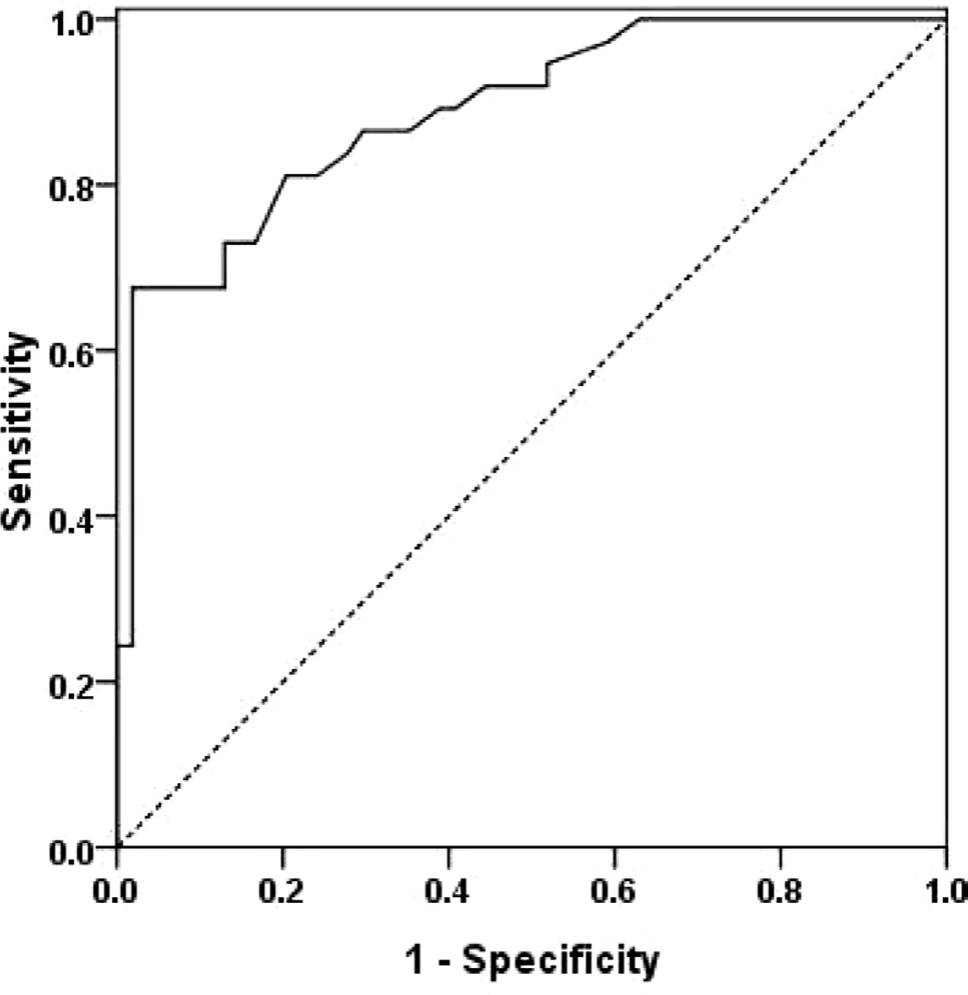
Receiver operation curve (ROC) of the multivariate logistic regression analysis model.

ROC was generated to examine the efficacy of the resulting model in predicting ischemic stroke or TIA recurrence (**Figure 2**). The area under the curve (AUC) was 0.888 (95% CI: 0.820–0.956, *P* < 0.05).

The inter-observer variability was 5% (1/20 × 100%). Thus, we can see that the uniformity between different doctors in judging the grade of plaque enhancement in CEUS is desirable.

## Discussion

High permeability of the neovessels plays an essential role in plaque growth. Furthermore, long-term presence of the neo-vessels can result in intraplaque hemorrhage and plaque progression, which increases the risk of ischemic stroke (Sun et al., 2016; Cheung et al., 2017).

TIA is considered to be an independent risk factor of ischemic stroke (Amarenco, 2018). Our study has demonstrated that with B-mode ultrasound, the proportion of unstable plaques detected was significantly higher in the recurrent group (17/37, 45.95%) compared to the non-recurrent group (10/54, 18.52%). This may suggest that the conventional ultrasonic features of instability were correlated with the occurrence of ischemic stroke. However, it is difficult to detect intraplaque neo-capillaries with conventional B-mode, color Doppler, or even power Doppler. Several studies (Huang et al., 2016; Amamoto et al., 2019) showed that CEUS had a high sensitivity in detecting neo-capillaries in carotid plaques. Based on a meta-analysis, the measurement of the degree of neovascularization was a promising tool in plaque evaluation (Huang et al., 2016). Unlike the contrast medium used in CT or angiography, the metabolic products of the contrast medium used in CEUS are exhaled from the lungs, such that the contrast medium is relatively safe. Based on the CEUS perfusion mode of neo-capillaries, the contrast enhancement of plaques was graded (Ott et al., 2008). Comparisons were made between post-endarterectomy carotid specimen and carotid arteries with different grades of contrast enhancement. Results showed a good correlation between the density of intraplaque microvessels and the grade of CEUS contrast enhancement. In addition, as for plaques with intraplaque hemorrhage, it has been proven that intraplaque liquefactive areas could be clearly shown and easily visible with CEUS, which may help in detecting unstable plaques (Varetto et al., 2015; Li et al., 2018).

The grading of intraplaque CEUS enhancement is based on the extent of ultrasonic contrast perfusion, which was strongly consistent with the density, course, and distribution of intraplaque capillaries. Results revealed that the proportion of Grade 3–4 contrast enhancement was apparently higher in the recurrent group (19/37, 51.35%) than in the non-recurrent group (12/54, 22.22%). This may suggest that the extent of intraplaque neoangiogenesis was strongly correlated with the risk of ischemic stroke. Moreover, multivariate logistic regression analysis has revealed that the grade of CEUS contrast enhancement was an independent risk factor for ischemic stroke or recurrent TIA. Hence, plaques with Grade 3–4 contrast enhancement would be more unstable than those with Grade 0–2 enhancement. Therefore, grade of CEUS contrast enhancement could become one of the predictive indices of ischemic stroke. For patients with a CEUS enhancement of Grade 3–4, immediate and effective treatment should be given for the secondary prevention. Besides, in this study, risk factors including diabetes mellitus and smoking history were not included in the logistic regression model. This may be due to the fact that our study was only restricted to individuals with TIA and the sample size was relatively small.

There are a number of limitations associated with our study. Firstly, as for patients with multiple plaques, only the largest of the plaques was selected in consideration of the image quality and dosage limitation of contrast media. This may introduce some potential sources of bias into the study. Secondly, the assessment of carotid plaques in this study was based on a semi-quantitative grading system rather than accurate quantitative measurement. The follow-up period was only 24 months; hence, a long-term follow-up is required in the following studies. Thirdly, this study was only restricted to individuals with TIA, and the sample size was relatively small; therefore, large, prospective multicenter studies are needed.

To summarize, our study provided evidence that CEUS could be a useful tool in detecting unstable plaques that were hard to assess by B-mode ultrasound. Perfusion mode of carotid plaques was an independent risk factor of ischemic stroke and recurrent TIA, which could be useful in guiding the prevention and treatment of the diseases in clinics.

## Ethics Statement

This study was carried out in accordance with the recommendations of institutional ethics committee of Shenzhen No.2 People’s Hospital, with written informed consent from all subjects. All subjects gave written informed consent in accordance with the Declaration of Helsinki. The protocol was approved by the institutional ethics committee of Shenzhen No.2 People’s Hospital.

## Author Contributions

ZL and XX contributed equally to this work. Other authors contributed equally to this work.

## Funding

This study received financial support from Shenzhen Science and Technology Project (project number JCYJ20170817171836611, JCYJ20170306092258717, and KJYY20180703165202011).

## Conflict of Interest Statement

The authors declare that the research was conducted in the absence of any commercial or financial relationships that could be construed as a potential conflict of interest.

The reviewer ML declared a shared affiliation, with no collaboration, with all of the authors to the handling editor at time of review.
